# Novel FGFR4-Targeting Single-Domain Antibodies for Multiple Targeted Therapies against Rhabdomyosarcoma

**DOI:** 10.3390/cancers12113313

**Published:** 2020-11-10

**Authors:** Nagjie Alijaj, Sandrine Moutel, Zelia L. Gouveia, Maxim Gray, Maurizio Roveri, Dzhangar Dzhumashev, Florian Weber, Gianmarco Meier, Paola Luciani, Jochen K. Rössler, Beat W. Schäfer, Franck Perez, Michele Bernasconi

**Affiliations:** 1 Department of Oncology, Children’s Research Center, University Children’s Hospital Zurich, 8032 Zurich, Switzerland; nagjielaila.alijaj@usz.ch (N.A.); maximgray@hotmail.co.uk (M.G.); maurizio.roveri@gmail.com (M.R.); beat.schaefer@kispi.uzh.ch (B.W.S.); 2 Department of Pediatric Hematology and Oncology, Inselspital, Bern University Hospital, 3010 Bern, Switzerland; dzhumashev.dzhangar@dbmr.unibe.ch (D.D.); jochen.roessler@insel.ch (J.K.R.); 3 Department for BioMedical Research (DBMR), University of Bern, 3008 Bern, Switzerland; 4 Institut Curie, PSL Research University, CNRS UMR144, 75248 Paris, France; sandrine.moutel@curie.fr (S.M.); zelia.gouveia@curie.fr (Z.L.G.); 5 Recombinant Antibody Platform (TAb-IP), Institut Curie, 75248 Paris, France; 6 Honing Biosciences, 75004 Paris, France; 7 Graduate School for Cellular and Biomedical Sciences, University of Bern, 3012 Bern, Switzerland; 8 Department of Chemistry and Biochemistry, University of Bern, 3012 Bern, Switzerland; florian.weber@dcb.unibe.ch (F.W.); paola.luciani@dcb.unibe.ch (P.L.); 9 Institute of Medical Microbiology, University of Zurich, 8006 Zurich, Switzerland; gmeier@imm.uzh.ch

**Keywords:** rhabdomyosarcoma, FGFR4, single-domain antibody, targeted liposomes, CAR T cells

## Abstract

**Simple Summary:**

Rhabdomyosarcoma (RMS) accounts for more than 50% of all soft tissue sarcomas in childhood and adolescence. Despite progress and intensified multimodality treatment, prognoses are extremely poor with an overall survival rate of approximately 20% in the advanced stage. Therefore, there is an urgent need for targeted treatment options to improve overall survival rates, and to limit long-term side effects. The fibroblast growth factor receptor 4 (FGFR4) is overexpressed in RMS and other tumors as well. The goal of this work was to select FGFR4 specific single-domain antibodies (sdAb) and to develop FGFR4-targeted therapies. We could show that FGFR4-targeted liposomes have the potential to deliver drugs specifically to FGFR4-positive tumor cells and that chimeric antigen receptor T cells built with the selected antibodies can kill specifically FGFR4-expressing RMS cells.

**Abstract:**

The fibroblast growth factor receptor 4 (FGFR4) is overexpressed in rhabdomyosarcoma (RMS) and represents a promising target for treatments based on specific and efficient antibodies. Despite progress, there is an urgent need for targeted treatment options to improve survival rates, and to limit long-term side effects. From phage display libraries we selected FGFR4-specific single-domain antibodies (sdAb) binding to recombinant FGFR4 and validated them by flow cytometry, surface plasmon resonance, and fluorescence microscopy. The specificity of the selected sdAb was verified on FGFR4-wild type and FGFR4-knock out cells. FGFR4-sdAb were used to decorate vincristine-loaded liposomes and to generate chimeric antigen receptor (CAR) T cells. First, incubation of RMS cells with FGFR4-sdAb revealed that FGFR4-sdAb can block FGF19-FGFR4 signaling via the MAPK pathway and could therefore serve as therapeutics for FGFR4-dependent cancers. Second, FGFR4-targeted vincristine-loaded liposomes bound specifically to RMS cells and were internalized by the receptor, demonstrating the potential for active drug delivery to the tumor. Third, FGFR4-CAR T cells, generated with one sdAb candidate, demonstrated strong and specific cytotoxicity against FGFR4 expressing RMS cells. We selected novel FGFR4-sdAb with high specificity and nano- to picomolar affinities for FGFR4 which have the potential to enable multiple FGFR4-targeted cancer therapy approaches.

## 1. Introduction

Approximately 50% of children and adolescents diagnosed with soft tissue sarcoma suffer from rhabdomyosarcoma (RMS), a striated-muscle lineage malignancy with variable pathologies [1]. The two major subtypes of the tumor are embryonal RMS (ERMS) and alveolar RMS (ARMS), accounting for 60% and 20% of all cases, respectively. ARMS is more aggressive and is characterized by a chromosomal translocation resulting in a *PAX3-FOXO1* gene fusion, whereas ERMS is associated with different tumor-promoting mutations and chromosome number aberrations [2]. Surgery, radiation, and multi-drug chemotherapy composed of vincristine (VCR), actinomycin D and cyclophosphamide are the standard treatments for RMS [1]. The overall survival rates for RMS patients have improved within the last few decades but the prognostic outcome is still very poor for high-risk patients, including those presenting metastatic diseases, ARMS subtype, or diagnosis in adulthood [3,4]. The treatment goes along with high toxicity and many who survive RMS will experience long-term adverse effects as adults [5]. Therefore, there is an urgent need for new targeted therapies to improve overall survival rates, and to overcome long-term side effects.

Nanovesicle-mediated chemotherapeutic drug delivery offers the possibility to increase the therapeutic effect in the tumor and to decrease side effects in healthy tissues [6,7,8,9]. Passive accumulation of nanoparticles in the tumors has been attributed to the so-called “enhanced permeability and retention” (EPR) effect [10]. Fast-growing solid tumors display a leaky vascular architecture and a lack of functional lymphatics, enabling the size-dependent passive extravasation and accumulation of nanoparticles in the interstitial space of the tumor [11]. Most recently, this dogma has been challenged by findings showing that the great majority of nanoparticles enter tumors using an active process through endothelial cells [12]. Liposomal formulations of chemotherapeutic drugs have demonstrated the safety and improved pharmacokinetic properties of the drug [13]. Prominent examples are liposomal doxorubicin (Doxil), daunorubicin (DaunoXome), and VCR (Marqibo) which have contributed to reducing side-effects compared to the free drug [14,15,16]. However, liposomal formulations have not, so far, been able to increase the therapeutic effect of the encapsulated drug. One possibility to achieve this is to modify the liposomal surface with tumor targeting-ligands, such as peptides [17], antibodies or antibody fragments [18], for active targeting to cancer cells. Single-domain antibodies (sdAb), first discovered in camelids [19], are the smallest possible antibody fragments (15 kDa) derived from heavy-chain antibodies. They are characterized by affinities comparable to conventional bivalent antibodies, as well as by high solubility, tissue penetration, and stability [20]. Previously, we developed the optimal formulation of liposomal VCR [21], and we investigated its pharmacokinetic and biodistribution in a mouse model engrafted with human RMS cells, revealing longer plasma circulation time and enhanced tumor accumulation of the liposomal drug compared to free VCR. Now, to further improve tumor accumulation of the liposomes in RMS by active targeting, we selected and investigated novel RMS-targeting sdAb.

The fibroblast growth factor receptor 4 (FGFR4) belongs to the family of receptor tyrosine kinases and is involved in myogenesis and muscle regeneration by promoting cell survival and differentiation [22,23]. FGFR4 is absent in normal differentiated muscles and is specifically overexpressed in RMS [24], as well as in other tumors, such as hepatocellular carcinomas, head and neck squamous cell carcinomas and basal-like breast cancer [25,26,27,28]. Therefore, FGFR4 represents a promising candidate for targeted therapies in RMS.

Another approach that could benefit from specific tumor targeting and that may improve the therapeutic outcome for RMS patients, is represented by chimeric antigen receptor (CAR) T cells. These cytolytic T cells are engineered with an extracellular antigen-binding domain recognizing specifically surface antigens on tumor cells. The intracellular part of the receptors is composed of T cell receptor signaling and costimulatory domains [29]. Tremendous clinical success has been achieved in the treatment of hematological malignancies with CAR T cells targeting CD19 [30,31], CD22 [32] and the B cell maturation antigen (BCMA) [33]. The application of CAR T cells for solid tumors has been more challenging, due to the lack of ideal tumor-specific target molecules, and also due to the strong immunosuppressive tumor microenvironment (TME) of solid tumors. Nevertheless, preclinical studies of CD276 (B7-H3) CAR T cells in pediatric solid tumors demonstrated good activity [34], and encouraging results have been reported for RMS CAR T cells targeting HER2 led to remission in a child with refractory metastatic RMS [35].

Here, we selected FGFR4-binding sdAb from two fully synthetic phage display libraries [36]. After validation, sdAb were coupled to the surface of VCR-loaded liposomes and tested as a potential drug-delivering platform for RMS cells in vitro. Moreover, we generated FGFR4 targeting CAR T cells with the selected sdAb and examined their cytotoxic potential for RMS cells in vitro.

## 2. Results

### 2.1. Phage Display Selection of FGFR4-Specific sdAb

**Figure 1:**
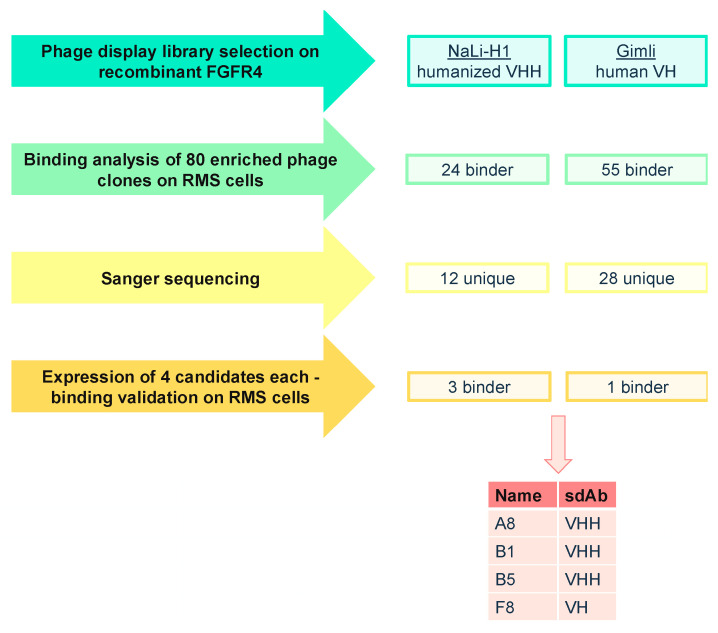
Schematic overview of phage display biopanning and preselection of FGFR4 binding single-domain antibodies (sdAb) sequences. Phage display selection was performed on biotinylated and Dynabeads-bound FGFR4 with two different synthetic sdAb phage display libraries. Enriched phage clones were tested for their binding to cell-surface FGFR4 on Rh4-FR4wt cells resulting in 40 unique binders. Eight sdAb were expressed in *E. coli* and purified. Of these, recombinant A8, B1, B5 and F8 bound to Rh4-FR4wt but not to Rh4-FR4ko cells.

Screening of FGFR4-binding sdAb was performed using two synthetic phage display libraries, the humanized VHH library NaLi-H1 [36] and the human VH library Gimli [37]. We performed two independent phage display selections with three rounds of biopanning against the recombinant extracellular portion of FGFR4 (Figure 1). In order to verify the binding specificity for FGFR4, we generated FGFR4 knockout RMS cells by CRISPR/Cas9 (Figure S1) and tested 80 phage clones from each screening for their binding to Rh4 FGFR4 wild-type cells (Rh4-FR4wt) and Rh4 FGFR4 knockout cells (Rh4-FR4ko). Flow cytometry analysis revealed 24 phage clones from NaLi-H1 library and 55 phage clones from Gimli library binding specifically to Rh4-FR4wt cells. Sanger sequencing of the 79 phage clones confirmed 12 unique sdAb from the NaLi-H1 library and 28 from the Gimli library. Next, four phage clones from each library (i.e., NaLi-H1: A8, B1, B5, C3; Gimli: A4, F8, F11, H2) that showed the strongest and most specific binding to Rh4-FR4wt by flow cytometry were expressed recombinantly. As negative control, we expressed an anti-mCherry sdAb (mCh) [36]. Recombinant sdAb were engineered to be expressed with a C-terminal Myc/6xHis-tag and an additional cysteine for maleimide coupling. 6xHis-tag purification and size exclusion chromatography resulted in proteins of high purity (Figure S4), with yields in the range of 3–16 mg per liter of bacterial culture. Hence, phage display selection resulted in eight sdAb candidates with potential binding to FGFR4.

### 2.2. Recombinant sdAb Bind to FR4wt RMS Cells But Not to FR4ko RMS Cells

**Figure 2:**
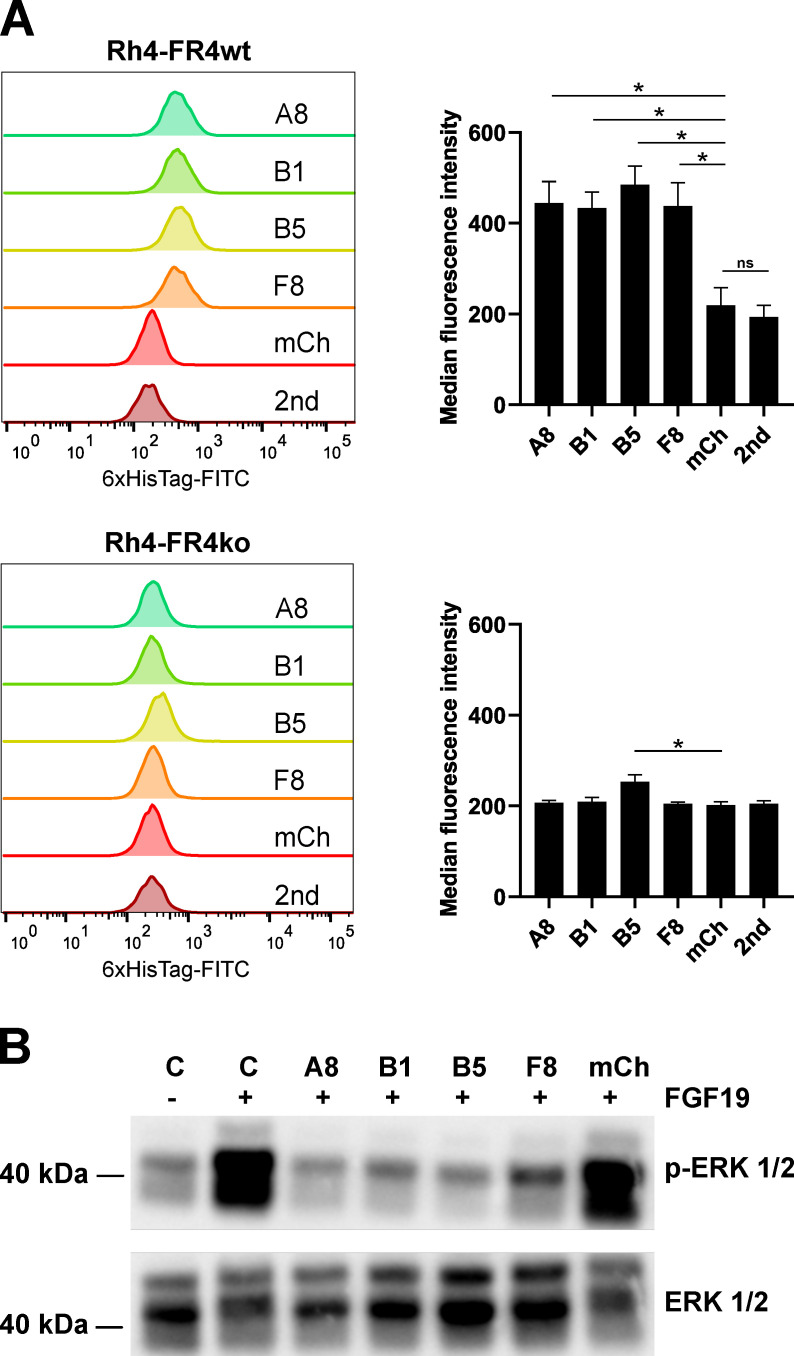
In vitro binding validation of sdAb. (**A**) SdAb were tested for their binding selectivity to cell surface FGFR4 by flow cytometry. Histograms show the single-cell living population of each sdAb binding to Rh4-FR4wt versus Rh4-FR4ko cells. Secondary FITC-labeled anti-6xHis-tag antibody alone (2nd) was used as background control and mCherry (mCh) was used as negative control. Median fluorescence intensities (MFI) were determined with FlowJoTM10 software. Statistics: paired t-test, *n* = 3 independent experiments, mean + SD, * *p* ≤ 0.05, ns = not significant. (**B**) Activation assay of FGFR4 in Rh30 cells was performed with recombinant FGF19 and in combination with sdAb. The cells were incubated for 1 h with sdAb at 10 μM (A8, B1, B5, F8, mCh) followed by stimulation of FGFR4 with 50 nM FGF19 for 10 min. Control cells were either not stimulated or stimulated with FGF19 in absence of the sdAb. The cell lysates were analyzed by Western blot with anti-phospho-ERK1/2 antibody. Total Erk1/2 levels are shown as loading control. The complete pictures of the Western blots can be found in Figure S3.

**Figure 3:**
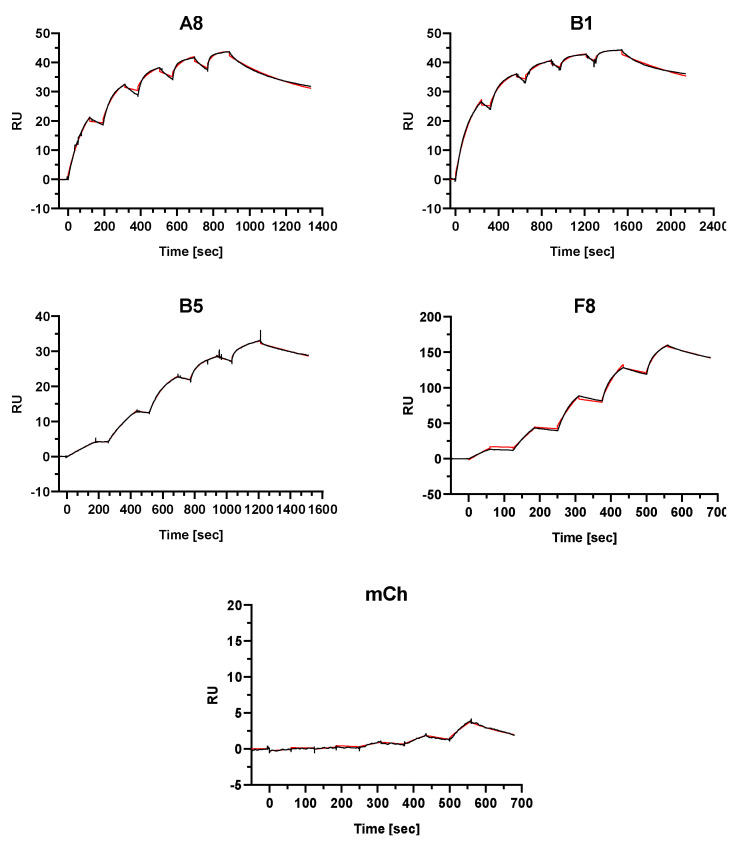
Affinity determination of sdAb to recombinant protein via surface plasmon resonance spectroscopy. Single-cycle kinetics analysis was performed on immobilized FGFR4 through covalent amine binding on the dextran-based sensor chip. The analytes A8, B1, B5, F8 and mCh were injected in 5 different concentrations followed by a dissociation phase. A final dissociation step was added after the last injection step to determine K_off_ rates for the KD calculations representing binding affinities. The black curves represent the measured data and red curves show the fit analysis (heterogeneous ligand model) performed with the BIAevaluation software.

Validation of the binding of the recombinant sdAb to cell-surface expressed FGFR4 was performed by flow cytometry on Rh4-FR4wt and Rh4-FR4ko cells. A fluoresceinisothiocyanat (FITC)-labeled anti-6xHis-tag antibody was used to detect surface-bound sdAb. Four of the recombinant sdAb tested displayed no binding to Rh4-FR4wt cells, whereas recombinant sdAb A8, B1, B5 and F8 showed significant binding to Rh4-FR4wt. A8, B1 and F8 did not bind to Rh4-FR4ko cells, while B5 showed a moderate binding to the knockout cells (Figure 2A). As expected, the anti-mCherry negative control sdAb did not bind to Rh4-FR4wt or to Rh4-FR4ko cells. Median fluorescence intensities (MFIs) of the four FGFR4 binders incubated with Rh4-FR4wt cells were in the range of 400, while the anti-mCherry negative control, as well as the anti-6xHis-tag antibody alone, only displayed MFI of 200. A similar value of about 200 was obtained when measuring the binding to Rh4-FR4ko cells, with a slightly higher value for B5. The extracellular domain of FGFR4 shares a high amino acid homology with FGFR1, 2 and 3 (55–72%). For the optimal targeting of RMS tumors, the aim is to identify binders specific for FGFR4 only. Rh4-FR4wt and Rh4-FR4ko cells both express FGFR1 and FGFR2, even though Rh4-FR4ko levels are slightly lower than Rh4-FR4wt (Figure S1). We concluded that the sdAb A8, B1 and F8 are specific for FGFR4 and do not bind to FGFR1 or FGFR2. Taken together, the binding validation of recombinant sdAb on RMS cells revealed four FGFR4-targeting sdAb candidates: A8, B1, B5 and F8. Since B5 showed a significantly higher MFI compared to mCh control on Rh4-FR4ko cells, its specificity to FGFR4 was further investigated (see below, Figure 3).

### 2.3. Selected Recombinant Single-Domain Antibodies Inhibit FGFR4 Signaling

Aberrant FGFR4 signaling is implicated in tumorigenesis of several cancers. FGFR4 initiates four major signaling pathways: RAS-MAPK, PI3K-AKT, PLCγ, and STAT [38]. We, therefore, tested the effect of the selected sdAb on FGFR4 activation and downstream signaling. FGFR4 activation assays were performed on Rh30 cells, an RMS cell line that has a low basal level of pERK and activates the MAPK pathway upon stimulation with the FGFR4-specific ligand FGF19. We incubated Rh30 cells with FGF19 with or without prior addition of sdAb (Figure 2B). As expected, the addition of FGF19 led to a drastic increase in phospho-ERK 1/2 levels. Remarkably, kinase activation was absent when Rh30 cells were preincubated with the selected sdAb, whereas negative control anti-mCherry did not block ERK 1/2 phosphorylation. These data showed that the selected sdAb A8, B1, B5, and F8 have the ability to block activation of the FGFR4 downstream MAPK pathway in RMS cells.

### 2.4. Single-Domain Antibodies Bind to FGFR4 with High Affinity

To determine the binding affinity of the sdAb to FGFR4, we performed surface plasmon resonance (SPR) spectroscopy using recombinant FGFR4 as a target. As mentioned above, FGFR1 and FGFR2 are expressed on Rh4-FR4ko cells and flow cytometry analysis indicated no binding of the sdAb to these cells. To further confirm FGFR4-specificity, we also performed affinity measurements using recombinant FGFR1, FGFR2, and FGFR3. SdAb A8, B1, B5, F8, and mCh were injected in five different concentrations in an FGFR-coated chip (Table S1). Except for the negative control mCh, calculated K_D_ values for FGFR4 binding were in the nano- and picomolar range (Figure 3, Table 1). Affinity parameters could not be fitted with a 1:1 binding model and best fits were obtained with the heterogeneous ligand model of the BIAevaluation software resulting in two K_D_ values for each candidate. Measurements performed using the receptor family isoforms FGFR1 and FGFR3 showed no binding of the analytes. We were only able to detect a measurable binding of A8 and B5 to FGFR2 in the order of micro- to nanomolar and nano- to picomolar, respectively (Figure S5; Table S2). The SPR data confirm the strong binding of all candidates to FGFR4 and suggests that only B1 and F8 have a strict FGFR4 specificity. Nevertheless, we decided to continue the characterization of all selected sdAb A8, B1, B5 and F8 for drug delivery and CAR T cell therapy.

### 2.5. Preparation and Characterization of VCR-Loaded Targeting Liposomes

**Figure 4:**
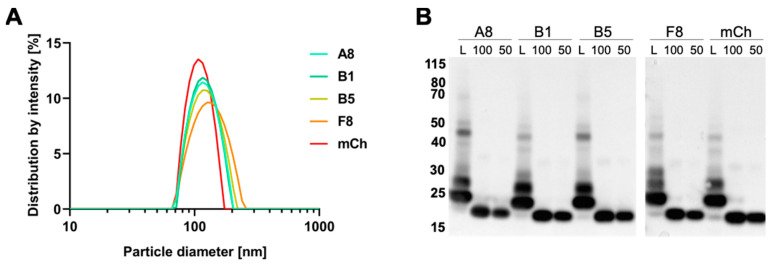
Characterization of vincristine-loaded targeted liposomes. (**A**) Size distribution of sdAb-coated liposomes measured by dynamic light scattering. (**B**) Western blot analysis of coupled sdAb. Liposome suspensions (L) equivalent to 100 ng of sdAb were loaded under reducing and denaturing conditions for gel electrophoresis. Amounts of 100 ng and 50 ng of uncoupled protein were loaded as control. SdAb were detected with an anti-6xHis-tag antibody.

In a previous study, we optimized the formulation of liposomal VCR [21]. Here, in order to optimize the conjugation to nanobodies, we introduced DSPE-PEG lipids with reactive maleimide groups at the distal end. SdAb harboring a free cysteine at the C-terminus were then coupled to the liposomal surface. Fluorescent liposomes composed of egg sphingomyelin, cholesterol, PEG-ceramide, DSPE-PEG-maleimide and the near-infrared dye DiR (49.8:45:4:1:0.2 mol%) were prepared by the film-hydration/extrusion method followed by VCR encapsulation and sdAb coupling. As expected, dynamic light scattering measurements of decorated liposomes L-A8, L-B1, L-B5, L-F8, and L-mCh revealed hydrodynamic diameters of approximately 120–135 nm and a monodisperse particle-size distribution characterized by a PDI of 0.03–0.13 (Figure 4A; Table 2).

The coupling of the sdAb to the liposomes was analyzed by Western blotting using an anti-6xHis-tag antibody. Samples of liposome suspensions were prepared with a theoretical sdAb amount of 100 ng. To estimate the coupling efficiency, we loaded 100 ng and 50 ng of the corresponding recombinant sdAb on the Western blot gel (Figure 4B). All liposome suspensions showed a dominant fraction running at an apparent size of 25 kDa, corresponding to one sdAb molecule (17 kDa) bound to DSPE-PEG-maleimide (2.9 kDa). Two further bands appear at a higher size suggesting the formation of complexes of two or three lipid molecules per sdAb. Besides the C-terminal cysteine, sdAb have two further cysteines forming an intramolecular disulfide bond and representing possible reaction sites for the maleimide groups. Notably, there was only a faint band corresponding to free sdAb in all the liposome samples.

To determine the encapsulation efficiency of VCR-loaded liposomes, an HPLC analysis was performed. The analysis showed a VCR encapsulation efficiency of approximately 97.8% with a final concentration of 250–320 μg/mL VCR in the liposomal formulations (Table 2). Thus, fluorescently labelled and sdAb-coated VCR-liposomes characterized by similar drug-loading, size and size-distribution profiles could be produced.

### 2.6. FGFR4-Targeted Liposomes Bind Specifically to FGFR4 Positive RMS Cells and Are Internalized

**Figure 5:**
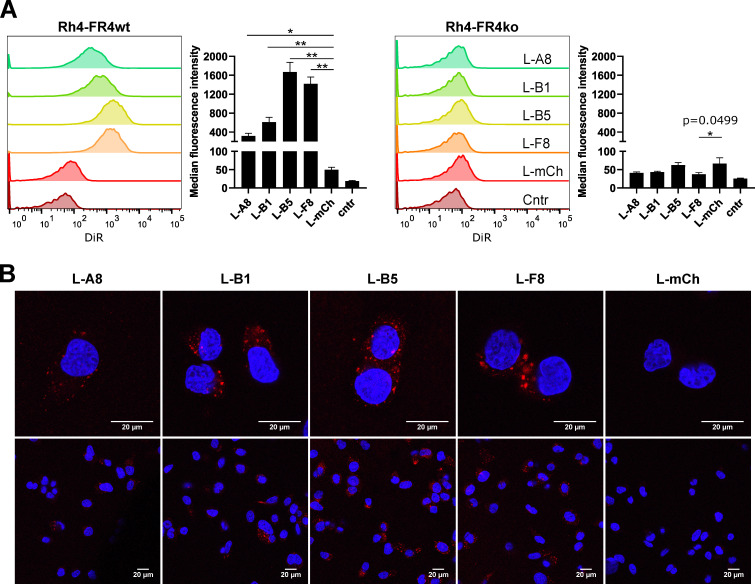
In vitro binding validation of FGFR4 targeting liposomes. (**A**). Liposomes decorated with FGFR4 targeting sdAb A8, B1, B5 and F8 or mCh (negative control) were tested for their binding selectivity to cell surface FGFR4 by flow cytometry. Adherent cells were incubated for 2 h with 0.5 mM total lipid concentration at 37 °C and 5% CO2. Histograms show the single-cell living population of liposomes binding to Rh4-FR4wt versus Rh4-FR4ko cells. Non-treated cells represent the control populations. Median fluorescence intensities (MFI) are shown. Statistics: paired *t*-test, *n* = 3 independent experiments, mean +SD, * *p* ≤ 0.05, ** *p* ≤ 0.01. (**B**) Confocal microscopy analysis of Rh4-FR4wt cells incubated for 2 h at 37 °C and 5% CO_2_ with sdAb coated fluorescent liposomes. The total lipid concentration was 3 mM. Cells were washed, fixed and mounted with DAPI containing medium. Internalization was visible only in Rh4-FR4wt cells incubated with FGFR4-targeted liposomes.

We next tested whether sdAb immobilized on the liposomal surface could still bind to FGFR4-expressing RMS cells. Rh4-FR4wt and Rh4-FR4ko cells were incubated for 2 h with fluorescent anti-FGFR4- or anti-mCherry-armed liposomes under normal cell culture conditions. DiR fluorescence was subsequently analyzed by flow cytometry (Figure 5A). Rh4-FR4wt cells incubated with FGFR4-targeted liposomes showed an increased fluorescent signal, indicating binding to FGFR4, while no increase in fluorescence was observed in Rh4-FR4ko cells incubated with FGFR4-targeted liposomes. Specifically, Rh4-FR4wt incubated with control mCherry-targeted liposomes had an MFI similar to non-treated cells, below 50. Among the FGFR4-targeted liposomes, MFI values ranged between 300, 600, 1700 and 1400 for L-A8, L-B1, L-B5 and L-F8, respectively, and were thus 6-, 12-, 34- and 28-fold higher than the MFI value of L-mCh (Figure 5A). These results show that sdAb linked to the liposomal surface are still able to bind specifically to Rh4-FR4wt when coupled to the surface of VCR-loaded liposomes, but binding intensities differed between the four sdAb.

Receptor-mediated internalization of drug-loaded liposomes increases intracellular drug amounts and thus enhances their therapeutic effect [39]. We, therefore, investigated the internalization of FGFR4-targeted liposomes by confocal microscopy. The liposomes were incubated for 2 h with Rh4-FR4wt and Rh4-FR4ko cells. Subsequently, images of the fixed cells revealed a clear intracellular uptake of all liposomes coated with FGFR4-targeting sdAb. Remarkably, no fluorescent signal was detected when Rh4-FR4wt cells were incubated with L-mCh (Figure 5B). Consistent with the flow cytometry data, L-A8 and L-B1 showed a weaker intracellular fluorescence. Strikingly, no fluorescence could be observed in Rh4-FR4ko cells, supporting their specificity for FGFR4 (Figure S6). Therefore, FGFR4-targeted liposomal formulations represent a specific drug-delivery platform for FGFR4 overexpressing RMS tumor cells, characterized by their rapid and specific receptor-mediated intracellular uptake.

### 2.7. Cytotoxicity of FGFR4-CAR T Cells Targeting RMS Cells

**Figure 6:**
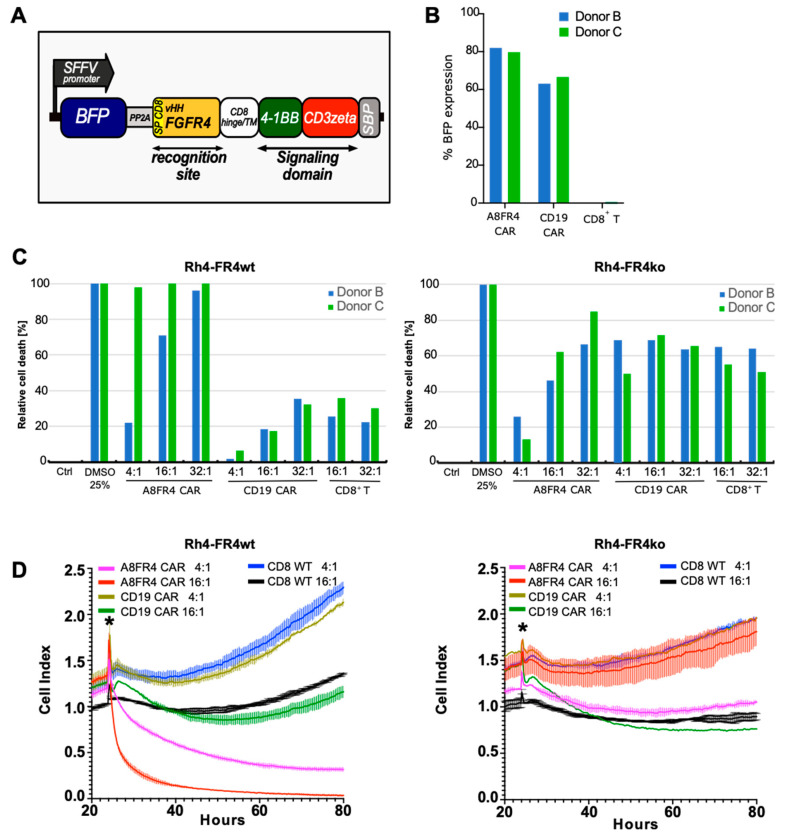
Cytotoxicity of FGFR4 chimeric antigen receptor (CAR) T cells towards Rhabdomyosarcoma (RMS) cells. (**A**) Schematic representation of the A8FR4 CAR construct. The CAR is composed of the sdAb A8 with CD8α signal peptide sequence and C-terminal myc-tag followed by the hinge and transmembrane (TM) domains of CD8α. Intracellular signaling domains are 4-1BB and CD3ζ and are followed by a streptavidin binding peptide (SBP). (**B**) CD8^+^ T cell transduction efficiencies of donor B and C were determined by flow cytometry analysis of BFP signal. (**C**) Cytotoxicity was determined by luciferase activity of Rh4 cells co-cultured for 72 h with effector T cells of donors B and C. Relative cell death was highest for Rh4-FR4wt cells incubated with A8FR4 CAR T cells at the indicated effector:target (E:T) cell ratios in both donors. In Rh4-FR4ko cells, non-specific cell killing was observed for the co-cultivation of all CAR T cells and the non-transduced CD8+ T cells. (**D**) Real-time cell death analysis of Rh4 cells co-cultured with effector T cells from donor B using xCELLigence RTCA DP. A8FR4-CAR T cells showed higher killing activity at the indicated E:T cell ratios in Rh4-FR4wt compared to non-specific CD19 CAR T cells or non-transduced CD8^+^ T cells. In Rh4-FR4ko cells, no specific cytotoxicity was observed. Horizontal lines within the curves indicate the SD of the duplicate wells used during the assay. The asterisks indicate the time of addition of the effector T cells.

To investigate the therapeutic potential of the selected sdAb, we decided to focus our attention on one selected sdAb, A8, to generate a chimeric antigen receptor (CAR) armed with anti-FGFR4 antibodies. The A8 sdAb was used to substitute the CD19 targeting single-chain antibody fragment (scFv) in a CD19-CAR T construct (CTL019/Kymriah, Novartis), currently used in hematologic cancer therapy [40]. The resulting A8_VHH_ FGFR4 CAR (A8FR4 CAR) is composed of the myc-tagged A8 followed by the hinge and transmembrane domains of CD8α and the intracellular signaling domains of 4-1BB and CD3ζ (Figure 6A). CD8^+^ T cells were isolated from four healthy donors (donor A, B, C and D) and were transduced with FGFR4 or CD19 targeting CARs. Transduction efficiency was measured by mTagBFP expression which showed about 80% A8FR4-CAR and 60% CD19-CAR positive cells (Figure 6B and Figure S7A). To assess the cytotoxic potency of the CAR T cells against Rh4-FR4wt and Rh4-FR4ko cells we applied bioluminescence and real-time cell death assays (Figure 6C,D). The RMS cells were co-cultured with CAR T cells at different ratios (E:T—Effector T cell to Target RMS cell), and as an additional control, we used non-transduced CD8^+^ T cells. The bioluminescence assay was performed with T cells from donor B and donor C and revealed the specific killing of Rh4-FR4wt by A8FR4-CAR T cells (Figure 6C). T cells from donor C showed higher cytotoxic efficiencies with almost 100% dead cells at the lowest E:T ratio of 4:1. The relative cell death of Rh4-FR4wt cells co-cultured with T cells from donor B at E:T ratios of 32:1, showed the selective cytotoxic effect of A8FR4-CAR T cells with almost 100% dead cells. CD19-CAR T cells and CD8^+^ control T cells reached only values of approximately 20–35% dead cells. T cell-mediated toxicity towards Rh4-FR4ko cells was similar for both CARs and CD8^+^ control T cells, although it was higher as compared to Rh4-FR4wt cells. The reasons for this unspecific sensitivity of Rh4-FR4ko cells to CAR Ts is unclear, it is not due to a difference in cell growth (Figure S2) but might be due either to knockout of FGFR4 or represent a clonal phenomenon. Real-time analysis of cell death with CARs from donors A, B and D showed similar results, with selective cell killing of Rh4-FR4wt by A8FR4-CAR T, but absent or reduced cytotoxicity in Rh4-FR4ko cells (Figure 6D and Figure S7B). Taken together, these data show that the selected sdAb A8 can generate CAR T cells targeting FGFR4 that mediate significant antitumor activity against FGFR4-expressing RMS cells in vitro and therefore represent a promising targeted treatment option. Studies on the other sdAb candidates B1, B5 and F8, and their potential in CAR T therapy, are currently ongoing.

## 3. Discussion

In this study, we developed three therapeutic strategies for RMS by targeting FGFR4 with sdAb and validated them on RMS cells. We selected four FGFR4 binding sdAb and tested them in vitro for (a) inhibitory activity of FGFR4 signaling; (b) active drug delivery as liposome conjugates, and (c) cell-mediated immunotherapy as CAR constructs.

The four selected sdAb A8, B1, B5 and F8 not only bind to FGFR4 expressed on RMS cells but are also able to block the FGF19-FGFR4-MAPK signaling axis. In ARMS, FGFR4 is a direct target gene of the fusion protein PAX3-FOXO1 [41], and in ERMS FGFR4 is frequently amplified with 12% of the tumors harboring activating mutations of the receptor [42,43,44]. In RMS, besides overexpression, FGFR4 has been shown to harbor activating mutations in over 6% of all tumors, resulting in constitutive tumor-promoting signaling within the cells [2,45,46]. Although we did not observe a toxic effect on cultured RMS cells, it is tempting to speculate that FGFR4 signaling could still represent a therapeutic target for sdAb in RMS. Moreover, FGFR4 is not only implicated in RMS tumorigenesis, but drives tumor progression in FGF19 expressing hepatocellular carcinomas, head and neck squamous cell carcinomas, and basal-like breast cancer [25,26,27,28]. It is also estimated that 0.5% of all tumors display abnormalities in FGFR4 [47]. The selected sdAb could therefore also serve as possible therapeutic approach for cancers other than RMS.

Surface plasmon resonance spectroscopy of sdAb binding to FGFR4 revealed strong affinities in the order of nano- to picomolar. The measured data could not be fitted with a 1:1 binding model. Best fits were obtained with the heterogeneous ligand model indicating two separate binding affinity parameters for the sdAb to FGFR4. We had to directly immobilize recombinant FGFR4 to activated carboxyl groups on the sensor chip through amine group binding. Binding of FGFR4 through a His-tag or biotin–streptavidin linker was not compatible with our measurements, since analytes and ligands contained a 6xHis-tag, and FGFR4 biotinylation led to interference with sdAb binding. Therefore, it is possible that the non-oriented binding of FGFR4 to the sensor chip could have led to a partial or complete steric hindrance of the sdAb binding site, resulting in heterogeneous binding parameters. This is obvious when comparing Rmax values, representing the maximal sdAb binding signal: for the affinity measurements of A8, we could immobilize 800 RU FGFR4 to the sensor chip. With approximate molecular weights of 40 kDa for the ligand and 17 kDa for the sdAb, we would expect an Rmax of 340 RU ((MWFGFR4/MWNB) × 800 RU) but ultimately a value of only 44 RU was achieved. Since we were not able to fully regenerate the flow cells after sdAb binding, we performed all measurements with freshly immobilized FGFR4 for each sdAb. This resulted in different amounts of immobilized FGFR4. A8, B1 and B5 analysis was performed with approximately 800 RU of FGFR4 whereas for F8 and mCh we immobilized 9000 and 12,000 RU, respectively. The measurement of negative control mCh on such high ligand densities forced unspecific interactions at high sdAb concentrations, and this resulted in low calculated affinities compared to the selected FGFR4 sdAb.

Both free and liposome-conjugated sdAb bound specifically to Rh4-FR4wt cells and showed, except for uncoupled B5 sdAb, no binding to Rh4-FR4ko. Nevertheless, recombinant B5 binding to Rh4-FR4ko cells in FACS experiments was only 0.25 times higher than mCh control sdAb, whereas on Rh4-FR4wt it was 2 times higher. Affinity measurements revealed binding of only A8 and B5 another FGFR-member, FGFR2. The binding affinity of A8 to FGFR2 was in the micromolar range and therefore very low when compared to the binding affinity to FGFR4. The fast koff rates further highlighted its weak binding to FGFR2. In contrast, B5 showed high affinities to FGFR2 in the low nanomolar range, thus similar to its affinity for FGFR4. Rh4 cells do express FGFR2, but protein levels are lower compared to FGFR4. Moreover, the Rh4-FR4ko cells have reduced FGFR1 and FGFR2 protein levels compared to Rh4-FR4wt. The reasons for the lower expression level of FGFR1 and FGFR2 in Rh4-FR4ko are not completely clear but could be due to a clonal effect or to regulatory loops. Therefore, it is well possible that the binding of sdAb A8 and B5 to cell surface FGFR2 would be only detectable above a certain expression level.

The formulation of liposomal VCR was modified from the previously established one [21] by the introduction of DSPE-PEG-maleimide at 1 mol%. As expected, the resulting physico-chemical properties of the liposomes and the drug loading efficiency were comparable. SdAb coupling to the surface was performed as described by Oliveira and colleagues [48] with 0.4 nmol sdAb per μmol of total lipids and it resulted in high coupling efficiencies. Among various conditions of the coupling reaction tested, we also tested higher sdAb-to-lipid ratios, but this resulted in precipitation of the liposomes. The fraction of uncoupled sdAb in the liposome suspension was negligible and it did not apparently interfere with binding on cells.

Confocal microscopy Rh4-FR4wt cells incubated with the fluorescent FGFR4-targeting liposomes showed a very specific internalization, represented by dot-like structures within the cells, which were absent in Rh4-FR4ko cells. The images were taken after 2 h of incubation, indicating a rather fast internalization process which can represent an advantage for a drug delivery platform to highly vascularized tumors.

In vitro cell assays are not ideal to predict and compare the in vivo therapeutic effects of drug-loaded targeting nanovesicles. Nonetheless, to verify if the increased binding and internalization observed by fluorescence analysis could translate into an increased activity of targeted VCR-loaded liposomes, we incubated RMS Rh4-FR4wt and Rh4-FR4ko cells with increasing concentrations of targeted liposomes and control L-mCh. We were not able to see significant differences in IC_50_ between the targeted liposomes and control, or between Rh4-FR4wt and Rh4-FR4ko cells, even after washing off the liposomes after 1 h or 2 h of incubation, to prevent any unspecific drug release during the three days of cultivation. The reasons for the lack of difference in activity between targeted and non-targeted liposomes are not clear. One hypothesis is that it might be due to unspecific binding of the liposomes to cell culture plates causing the release of the cytostatic content. Therefore, the therapeutic potential of FGFR4-targeted drug delivery to RMS needs to finally be evaluated in an RMS in vivo model.

Importantly, we were able to verify the selective cell-mediated cytotoxicity of sdAb-based FGFR4 CAR T cells towards Rh4-FR4wt. Although we observed some differences in cytotoxic efficiencies between three CD8+ T cell donors, all FGFR4-CAR Ts showed the same specific trend. Real-time cell analysis represents an elegant tool to monitor the cytotoxic potential of CAR Ts and revealed no or lower effects of FGFR CAR Ts on Rh4-FR4ko, comparable to that of control CD19 CAR Ts. We believe that the immune-based treatment of RMS with FGFR4 CAR Ts holds promising potential, since RMS tumors display aberrantly high FGFR4 expression compared to healthy tissues [42]. It has been shown that high antigen densities above a certain threshold level are required for effective CAR T cell activation, offering a therapeutic window for RMS treatment [49,50]. Further studies will be required to test FGFR4 CAR Ts efficiency in a RMS in vivo model.

## 4. Materials and Methods

### 4.1. Plasmids and Cloning

For recombinant protein expression, sdAb encoding sequences on the pHEN2 phagemid vector were PCR amplified with SapI-introducing primers for FX cloning [51] into pSB_initC (kindly provided by M. Seeger lab, University of Zurich, Zurich, Switzerland). The expression vector harbors a ccdB suicide cassette, a C-terminal cysteine followed by Myc-tag and 6xHis-tag. Successful cloning of sdAb sequences replaced ccdB and the constructs were amplified in *E. coli* MC1061. CAR T cell constructs were generated with the A8 sdAb sequence and were cloned by ligation into the pTRIP-BFP-2a-scFvCD19-myc-41BB-CD3ζ-SBP with the substitution of the scFvCD19 by A8 (pTRIP-BFP-2a-vHH-FGFR4-myc-41BB-CD3ζ-SBP). The pTRIP-BFP-2a-scFvCD19-myc-41BB-CD3ζ-SBP was previously generated by gene synthesis of the sequence composed of: Single peptide CD8α/Single-chain variable fragment against CD19/Myc tag/CD8α hinge and transmembrane domain/Stimulatory domains 41BB and CD3ζ/Streptavidin binding peptide (SBP). This gene was cloned into the pTRIP-SFFV-tagBFP-2A kindly provided by Nicolas Manel (Institut Curie, Paris, France) [52].

### 4.2. Cell Lines

The cell lines Rh4 (kindly provided by Peter Houghton, Research Institute at Nationwide Children’s Hospital, Columbus, OH, USA), Rh30, HEK293ft, HEK293T (purchased from ATCC, LGC Standards S.a.r.l, Molsheim, France) were maintained in DMEM supplemented with 10% FBS (both Sigma-Aldrich, Buchs, Switzerland), 2 mM L-glutamine and 100 U/mL penicillin/streptomycin (both from Thermo Fisher Scientific, Illkirch, France) at 37 °C in 5% CO_2_. RMS cell lines were tested and authenticated by cell line typing analysis (STR profiling) in 2014/2015 and positively matched [53]. All cell lines tested negative for mycoplasma.

### 4.3. Generation of CRISPR/Cas9 FGFR4 Knockout Cells

Rh4 FGFR4 knockout clones were generated via CRISPR/Cas9 technology. Complementary single-strand oligonucleotides encoding the sgRNA sequence for FGFR4 knockout (TTGCACATAGGGGAAACCGT) were annealed and cloned into the lentiCRISPRv2 puro vector (#98290, Addgene) via Esp3I (ER0451, Thermo Fisher Scientific, Illkirch, France) restriction and T4 ligation (15224017, Thermo Fisher Scientific, Illkirch, France). Lentiviral vectors were produced in HEK293T cells. The cells were transiently transfected with pMDL, pREV, pVSV-G and the lentiCRISPRv2-sgFR4Ex14 using JetPrime (Polyplus Transfection, Illkirch-Graffenstaden, France). After 24 h, medium was replaced, and virus supernatant was harvested after additional 48 h. The supernatant was filtered, 20-fold concentrated (Amicon Ultra 15, Merck Millipore, Schaffhausen, Switzerland; 4000× *g*, 15 min) and stored at −80 °C. Transduction of RMS cells was performed with concentrated viral supernatant in the presence of 10 µg/mL polybrene (Merck Millipore, Schaffhausen, Switzerland). After 24 h, medium was changed and puromycin selection at 1 µg/mL was started after 72 h and carried out for 7 days. Single-cell cloning was performed with selected cells on 96-well plates and the FGFR4 knockout was confirmed on protein level by Western blotting. All experiments were performed with the knockout clone #8.

### 4.4. Production of Lentiviral Vector for CAR T Cell Construction

Lentivirus particles were produced by co-transfection of the plasmid containing the genes of interest (BFP-2a-scFvCD19/VHH-FGFR4-myc-41BB-CD3ζ-SBP), the packaging plasmid psPAX2 and envelop plasmid pVSV-G into HEK293ft using the polyethyleneimine (PEI) precipitation protocol. The cells were incubated at 37 °C with 5% CO_2_ and the supernatant was harvested after 48 h and 72 h, pooled and filtered using a 0.45 μm filter. To concentrate the lentivirus particles, 20% sucrose in PBS was applied to the filtered supernatant followed by centrifugation at 100,000× *g* for 1.5 h at 4 °C. The pellet was recovered in 1 mL of freezing medium (DMEM complete medium + 0.1 mM β-mercaptoethanol (Gibco, Thermo Fisher Scientific, Illkirch, France) and 1 mM HEPES (Gibco, Thermo Fisher Scientific, Illkirch, France) and stored at −80 °C until use. Lentivirus titer was determined by flow cytometry through the detection of fluorescent protein (mTagBFP) in HEK293ft cells 72 h after transduction.

### 4.5. T Cell Isolation and Transduction

Peripheral blood mononuclear cells (PBMCs) were recovered using the density gradient Lymphoprep (StemCells, Grenoble, France). CD8^+^ T cells were isolated by negative selection using the CD8^+^ T cell human isolation kit (Miltenyi Biotec, Paris, France). Isolated CD8^+^ T cells were then cultured in X-VIVO medium (Lonza, Colmar, France) supplemented with 50 μM of β-mercaptoethanol (Merck Millipore, Schaffhausen, Switzerland) and 5% human serum (Merck Millipore, Schaffhausen, Switzerland) and activated using human T-activator CD3/CD28 Dynabeads (Gibco, Thermo Fisher Scientific, Illkirch, France). Approximately 24 h after T cell activation, the T cells were transduced with lentiviral particles mixed with 4 μg/mL of polybrene (Merck Millipore, Schaffhausen, Switzerland) at an MOI of 5 or higher. Two days after, the medium was exchanged and replaced by fresh medium supplemented with 5 ng/mL recombinant human interleukin-2 (IL2; R&D Biosystem, Bio-Techne SAS, Noyal Châtillon sur Seiche, France). The transduction efficiency was evaluated at day 6 or 7 after transduction through the detection of mTagBFP expressing cells using flow cytometry. The healthy adult blood donors (Saint-Louis Etablissement Français du sang (EFS) or Saint-Antoine Crozatier EFS at Paris, France) consented to provide their blood for research purposes.

### 4.6. Phage Display Selection

Screening for FGFR4 binding sdAb was performed with biotinylated extracellular FGFR4 (G&P Biosciences, Santa Clara, CA, USA) in native condition as described [54] using Nali-H1 library [36] composed of 3 × 10^9^ synthetic humanized VHH and Gimli library [37] composed of 1.6 × 10^9^ synthetic human VH.

### 4.7. Protein Expression and Purification

Periplasmic expression of sdAb was performed in E. coli MC1061 harboring the pSB_init vector enabling protein production with a C-terminal cysteine and 6xHis-tag. A 20 mL overnight pre-culture grown in Terrific Broth medium (25 µg/mL Chloramphenicol) was diluted in 2000 mL fresh medium and grown at 37 °C for 2 h. The temperature was then reduced to 25 °C and after 1 h protein expression was induced with 0.02% L-arabinose. The bacterial culture was grown overnight at 25 °C and cells were harvested by centrifugation (12,000× *g*, 15 min). Periplasmic protein extraction was performed with the osmotic shock method. The cells were resuspended with 50 mL lysis buffer 1 (50 mM Tris/HCl, pH 8.0, 20% sucrose, 0.5 mM EDTA, 5 μg/mL lysozyme, 2 mM DTT) and incubated for 30 min on ice. After the addition of ice-cold lysis buffer 2 (PBS, pH 7.5, 1 mM MgCl_2_, 2 mM DTT), the cell debris was harvested by centrifugation (3800× *g*, 15 min) and the protein-containing supernatant was supplemented with a final concentration of 10 mM imidazole. Ten mL of Co^2+^-beads slurry (HisPur Cobalt Resin, Thermo Fisher Scientific, Illkirch, France) were washed with wash buffer (PBS, pH 7.5, 30 mM imidazole, 2 mM DTT) and the supernatant was added to the beads. After an incubation of 1 h at 4 °C, the beads were washed with 20 mL wash buffer and bound protein was eluted with 20 mL elution buffer (PBS, pH 7.5, 300 mM imidazole, 2 mM DTT). Prior size exclusion chromatography (SEC), with a Sepax SRT-10C SEC100 column (Sepax Technologies, Newark, DE, USA) equilibrated with PBS, pH 7.5, 2 mM DTT, the protein elution was dialyzed overnight into PBS, pH 7.5, 2 mM DTT and concentrated via spin filter centrifugation (Amicon Ultra 15, 3 kDa, Merck Millipore, Schaffhausen, Switzerland).

### 4.8. Flow Cytometry

Binding validation of selected phages, recombinant sdAb and decorated liposomes was performed on Rh4-FR4wt and Rh4-FR4ko cells. The specificity of selected phage clones binding to FGFR4 was determined by flow cytometry in 96-well plates (BD Biosciences, Le Pont de Claix, France). Cell surface staining of Rh4-FR4wt or Rh4-FR4ko cells was performed on ice in PBS supplemented with 1% FBS. Eighty µL phages + 20 µL PBS/1% milk were incubated on 1 × 10^5^ cells for 1 h on ice. After 2 washes in PBS, phage binding was detected by a 1:250 dilution of anti-M13 antibody (27-9420-01; GE healthcare, Buc, France) for 1 h on ice followed by a 1:400 dilution of A488-conjugated anti-Mouse antibody (715-545-151; Jackson ImmunoResearch, Europe Ltd., Ely, UK) for 45 min. Samples were analyzed after two washes by flow cytometry on a MACSQuant cytometer (Miltenyi, Biotec, Paris, France) and results were analyzed with FlowJo software (BD Biosciences, Le Pont de Claix, France). Phages displaying anti-mCherry sdAb were used as a negative control [36]. For binding tests of recombinant sdAb, cells were detached with Accutase (Stemcell Technologies, Grenoble, France) and washed with PBS. All following steps were performed on ice: 4 × 10^5^ cells were incubated with sdAb concentrations of 30 µg/mL for 1 h, washed once with PBS and incubated for an additional 30 min with anti-6xHis-tag FITC-labeled antibody (LS‑C57341, LSBioscience, LabForce. Muttenz, Switzerlnad, diluted 1:10). The cells were washed once more with PBS and analyzed. Targeting liposomes were added at 0.5 mM final lipid concentration in complete medium to cells in 96-well plates and incubated for 2 h at 37 °C and 5% CO_2_. The cells were washed twice with PBS and detached with Accutase. All flow cytometry measurements were performed with Fortessa flow cytometer (BD Biosciences, Allschwil, Switzerland) and the data were analyzed using FlowJo^TM^ 10.4.1 software (Becton, Dickinson & Company, Franklin Lakes, NJ, USA). Statistical analysis was performed with GraphPad Prism, version 8 (San Diego, CA, USA). The statistical difference was assessed by paired t-test and *p* ≤ 0.05 was considered as statistically significant.

### 4.9. FGFR4 Activation Assay

To test the effect of sdAb on FGFR4 activation, 6 × 10^4^ Rh30-FR4wt and Rh30-FR4ko cells were plated on 24-well plates. The next day, sdAb were added at 10 µM concentrations to the cells in FBS-free medium and incubated for 1 h at 37 °C prior to stimulation with 50 nM recombinant human FGF19 (Peprotech, Lubio science, Zurich, Switzerland) for 10 min. Cells were immediately washed with ice-cold PBS and lysed in Tris/RIPA buffer (50 mM Tris HCl, pH 7.5, 150 mM NaCl, 1% NP40, 0.5% Na-Deoxycholate, 0.1% SDS, 1 mM EGTA, with standard protease and phosphatase inhibitors). Total cell extracts were then analyzed by Western blotting.

### 4.10. Western Blotting

SDS-PAGE samples were separated on 4–12% NuPAGE Bis-Tris gels (Thermo Fisher Scientific, Illkirch, France) and blotted on Trans-Blot Turbo Transfer Blot membranes (Biorad, Cressier, Switzerland). After blocking the membranes with blocking buffer (5% milk/TBST) for 1 h at room temperature, the primary antibody was added at a 1:1000 dilution and incubated overnight at 4 °C. The secondary HRP-conjugated antibody was diluted 1:10,000 in blocking buffer and added to the washed membrane for 1 h at room temperature. Chemiluminescence was detected after incubation with Amersham^TM^ ECL^TM^ detection reagent (GE Healthcare, Opfikon, Switzerland) or SuperSignal^TM^ West Femto Maximum Sensitivity Substrate (Thermo Fisher Scientific, Illkirch, France) in a ChemiDoc^TM^ Touch Imaging System (BioRad, Cressier, Switzerland). Primary antibodies used were p44/42 MAPK ERK1/2 (#9102), phospho-p44/42 MAPK Thr202/Tyr204 (#9101), β-Tubulin D3U1W (#86298), FGF Receptor 1 D8E4 (#9740) (all from Cell Signaling Technology, BioConcept, Allschwil, Switzerland), FGF Receptor 2 C-17 (sc-122), FGF Receptor 3 B9 (sc-13121) and FGF Receptor 4 A-10 (sc-136988) (all from Santa Cruz Biotechnology, LabForce, Muttenz, Switzerland). Secondary antibodies were anti-rabbit IgG (#7074, Cell Signaling Technology, BioConcept, Allschwil, Switzerland) and anti-mouse IgG (#7076, Cell Signaling Technology, BioConcept, Allschwil, Switzerland).

### 4.11. Surface Plasmon Resonance Spectroscopy

Single-cycle kinetics analysis was performed with the BIAcore T200 instrument (GE Healthcare, Opfikon, Switzerland) on CMD200M sensor chips (XanTec bioanalytics GmbH) activated with a mixture of 300 mM NHS (*N*-hydroxysuccinimide) and 50 mM EDC (*N*-ethyl-N’-(dimethylaminopropyl) carbodiimide). Recombinant FGFR1, FGFR2, FGFR3 and FGFR4 (G&P Biosciences) were immobilized on the activated biosensors (800 to 12,000 RU; 1 RU = 1 pg/mm^2^) followed by a blocking step with 1 M ethanolamine. One flow channel per chip was used as a reference to provide background corrections. The sdAb were injected at 5 different concentrations followed by a dissociation phase. All injections and washing steps were performed with TBST buffer. K_off_-rates were determined from a final dissociation step after the last injection. The measurements with FGFR4 were performed for each sdAb on freshly immobilized protein due to strong binding and incomplete dissociation from the surface. The immobilization flow rate was 5 μL/min and binding studies were performed at 30 μL/min. Binding parameters were determined with the heterogeneous ligand model fit of the BIAevaluation software.

### 4.12. Preparation of Fluorescently Labelled VCR-Loaded Liposomes

The production of liposomes and vincristine (VCR) loading was performed as described [21], with minor modifications. Liposomes were produced with the film-hydration/extrusion method with egg sphingomyelin (Lipoid GmbH, Ludwigshafen am Rhein, Germany), cholesterol (Sigma Aldrich, Buchs, Switzerland), PEG-ceramide (*N*-palmitoyl-sphingosine-1-[succinyl [methoxyPEG-2000]]), DSPE-PEG-maleimide (1,2-distearoyl-sn-glycero-3-phosphoethanolamine-*N*-[maleimide (polyethylene glycol)-2000]) (both Avanti Polar Lipids, Alabaster, AL, USA) and DiR (1,1′-dioctadecyl-3,3,3′,3′-tetramethylindotricarbocyanine Iodide) (Thermo Fisher Scientific, Illkirch, France) at a ratio of 49.8:45:4:1:0.2 mol%, respectively. The lipid film was hydrated with citrate buffer (250 mM, pH 3) resulting in a multilamellar liposomal dispersion having 70 mM of total lipid concentration. Next, six freeze-thaw cycles and ten extrusion steps with a LIPEX^®^ Thermobarrel extruder (Evonik Nutrition and Care GmbH, Essen, Germany) and a 100 nm pore-size polycarbonate membrane (Whatman, Maidstone, UK) were performed. A transmembrane pH gradient was generated via gel exclusion chromatography with PD MidiTrap™ Sephadex G-25 columns (GE Healthcare, Opfikon, Switzerland). The columns were conditioned with coupling buffer (PBS, pH 7.0) and the eluted liposome suspensions (14 mM) were used for VCR encapsulation. For a molar drug-to-lipid ratio of 0.05, 1 mL of liposomes were mixed with 1 mL of 0.7 mM VCR (VincristineTeva, Teva Pharma AG, Basel, Switzerland) diluted in coupling buffer and incubated for 1 h at 65 °C. Non-encapsulated VCR was removed via spin filter centrifugation (Amicon Ultra 0.5, 100 kDa, Merck Millipore, Schaffhausen, Switzerland). Final VCR-loaded liposomes preparations had a total lipid concentration of 11.2 mM.

### 4.13. Decoration of Liposomes with sdAb

For coupling of the sdAb to the liposomal surface, the buffer was exchanged to coupling buffer (PBS, pH 7.0) with PD MiniTrap™ Sephadex G-25 columns (GE Healthcare, Opfikon, Switzerland). A sdAb to lipid ratio of 0.4 nmol/μmol was chosen for the reaction, resulting in approximately 30 sdAb per liposome, as described previously [48]. The reaction was incubated overnight at 4 °C and non-coupled sdAb were removed by two steps of washing and filtration via spin filter centrifugation (Amicon Ultra 0.5, 100 kDa, Merck Millipore, Schaffhausen, Switzerland). The mean diameter and polydispersity index (PDI) of liposomes were measured by dynamic light scattering (Litesizer 500, Buchs, Switzerland). To estimate the amount of sdAb coupled to the liposomes, gel electrophoresis was performed with labelled liposomes and defined amounts of the corresponding sdAb under denaturing and reducing conditions. Sample separation, Western blotting and imaging were performed as described above with anti-6xHis-tag antibody (ab 18184, Abcam, Cambridge, UK).

### 4.14. VCR Quantification

Quantification of VCR concentrations was performed via HPLC (Ultimate 3000 HPLC system equipped with a DAD-3000 diode array detector, Thermo Fisher Scientific, Illkirch, France) with an RP-18 (5 μm, 4.6 × 250 mm) LiChrospher^®^ 100 column (Merck Millipore, Schaffhausen, Switzerland), optimizing a previously reported method [21]. A calibration curve for VCR ranging from 890 µg/mL to 13.9 µg/mL was prepared and liposome samples were disrupted with methanol for analysis. Doxorubicin was mixed to all samples to a final concentration of 50 µg/mL, serving as an internal standard. A di-potassium phosphate buffer (50 mM, pH 3.2) was used as mobile phase (68%) with a mixture of acetonitrile/UPW 90/10 (*v/v*; 32%) for 30 min at a flow rate of 1.5 mL/min. For each sample, a volume of 20 μL was injected. VCR and doxorubicin were detected at λ = 230 nm. Drug-loading efficiency was determined by analyzing VCR concentrations in the spin-filter purified liposome suspension and the aqueous flow-through. The encapsulation efficiency represented the percentage of VCR in the liposome suspension compared to the combined amount of VCR from filtered liposomes and flow-through. 

### 4.15. Confocal Microscopy

Detection of binding and internalization of fluorescent liposomes was performed on Rh4 wildtype and Rh4-FGFR4-knockout cells via confocal laser scanning microscopy (CLSM-Leica SP8 inverse, Heerbrugg, Switzerland). An amount of 40,000 cells were seeded in a four-well microscopy slide (Falcon™ Chambered Cell Culture Slides, Fisher Scientific, Illkirch, France). The next day, targeted or control liposomes were added to the cells at a final lipid concentration of 3 mM, and incubated for 2 h at 37 °C and 5% CO_2_. The wells were then washed twice with PBS and the cells were fixed for 15 min with 4% formaldehyde solution. After two further washing steps with PBS, the slides were separated from the chamber case and mounted with DAPI-containing medium (VECTASHIELD^®^ Hardset Antifade Mounting Medium with Phalloidin, Vector Laboratories, Adipogen AG, Liestal, Switzerland). Microscopy imaging was performed with 63x objective (HC PL APO CS2 63x/1.30) and the lasers Diode405 and Diode638 for DAPI and DiR excitation, respectively. All images were processed with ImageJ (v1.52s).

### 4.16. CAR T Cell Cytotoxicity Assays

Two methods were used to evaluate the cytotoxicity of T cells toward RMS cells. For the bioluminescence assay, Rh4-FR4wt and Rh4-FR4ko cells were transduced with lentiviral particles to express both mTagBFP and Red Firefly luciferase using a P2A fusion. In addition, a fluorogenic reporter YFAST [55] fused to puromycin resistance gene was expressed using an EF1 promoter (BFP-P2A-Luciferase-pEF1-YAST-Puromycin). Briefly, 4000 target cells were plated in a 96-well ViewPlate Black (Perkin-Elmer, Villebon-sur-Yvette, France) in complete DMEM medium and effector cells (CD8^+^ T cells) were added the next day at the indicated effector to target (E:T) ratios in X-VIVO medium (2-fold volume compared to DMEM). After approximately 72 h of incubation at 37 °C and 5% CO_2_, the wells were washed twice with PBS and 1–2 mg/mL of luciferin substrate (Perkin Elmer, Villebon-sur-Yvette, France) in PBS was added for 10 min (37 °C) prior to luminescence measurement with FLUOstar OPTIMA (BMG LabTech, Champigny-sur-Marne, France). The percentage of cell survival was calculated by taking the luminescence values for each point and dividing it by the highest value of luminescence obtained. Real-time cell death measurements were performed with the xCELLigence Real-Time Analyzer System (ACEA Biosciences, San Diego, CA, USA). Briefly, 10,000 target cells were plated in a 16-well E-plate (ACEA Biosciences, San Diego, CA, USA) in complete DMEM medium and the next day the effector cells were added at indicated E:T ratios in X-VIVO medium (2-fold volume compared to DMEM). Cell index (relative impedance) was monitored in real-time every 15 min for about four days at 37 °C and 5% CO_2_.

## 5. Conclusions

We selected FGFR4-specific sdAb with inhibitory effects on receptor signaling, which allowed us to develop an efficient drug-delivery platform for the treatment of FGFR4 positive tumors via targeted liposomes. Moreover, we could achieve effective cell-mediated cytotoxicity with FGFR4-CAR T cells in vitro. Therapies for RMS based on FGFR4 antibodies have been investigated pre-clinically with promising results, either as antibody–drug conjugates (ADC) [56,57,58], or with the antigen-binding domain grafted on chimeric antigen receptors (CARs) to generate CAR T cells [59]. With our work, we show here a novel FGFR4-targeting based on sdAb by active drug delivery and T cell recruitment. These tumor-targeting approaches could be further applied to other FGFR4-expressing tumors, such as hepatocellular carcinomas, head and neck squamous cell carcinomas, and basal-like breast cancer. 

## 6. Patents

N.A., S.M., Z.L.G., M.B. and F.P. filed on 20 May 2020 a European Patent Application number EP20305535 entitled «Single-domain antibodies and their use in cancer therapies» to protect commercial use of the antibodies described here.

## Figures and Tables

**Table 1:** Surface plasmon resonance spectroscopic determination of sdAb binding affinities to FGFR4.

SdAb	k_on_1 * (1/M × s)	k_off_1 (1/s)	K_D_1 (M)	k_on_2 (1/M × s)	k_off_2 (1/s)	K_D_2 (M)	R_max_1 ** (RU)	R_max_2 (RU)
A8	3.14 × 10^5^	1.32 × 10^−9^	4.22 × 10^−15^	6.67 × 10^4^	2.45 × 10^−3^	3.68 × 10^−8^	25.6	18.1
B1	1.11 × 10^6^	1.18 × 10^−6^	1.06 × 10^−12^	2.16 × 10^5^	9.92 × 10^−4^	4.60 × 10^−9^	26.1	17.5
B5	1.84 × 10^6^	5.66 × 10^−4^	3.08 × 10^−10^	1.73 × 10^5^	3.75 × 10^−8^	2.16 × 10^−13^	23.1	10.3
F8	5.45 × 10^4^	1.04 × 10^−6^	1.91 × 10^−11^	1.35 × 10^6^	5.57 × 10^−3^	4.14 × 10^−9^	83.0	86.4
mCh	2.60 × 10^3^	5.11 × 10^−3^	1.96 × 10^−6^	2.32 × 10^3^	5.05 × 10^−3^	2.18 × 10^−6^	20.7	20.7

* Measured data were fitted with the heterogeneous ligand model and revealed association and dissociation constants (k_on_ and k_off_) used for calculating affinities in terms of dissociation equilibrium constants KD (k_off_/k_on_). ** The maximal analyte binding signal R_max_ is indicated in RU for both determined KD and resembles their fraction within the amount of total bound sdAb.

**Table 2:** Characterization of FGFR4-targeting and vincristine (VCR)-loaded liposomes.

Liposomes Characteristics	L-A8	L-B1	L-B5	L-F8	L-mCh
Hydr. diameter (nm)	126	127	129	136	122
PDI	0.129	0.108	0.093	0.122	0.025
VCR conc (µg/mL) ^*^	308.4	253.8	254.6	270.3	319.9

* VCR end concentrations of the five formulations were determined by HPLC.

## Supplementary Materials

The following are available online at https://www.mdpi.com/2072-6694/12/11/3313/s1, Figure S1: FGFR protein levels in Rh4 cells, Figure S2: Real-time cell analysis of Rh4-FR4wt and Rh4-FR4ko cells growth, Figure S3 Original unmodified pictures of Western blot shown in Figure 2B, Figure S4: Purification of recombinantly expressed sdAb, Figure S5: Surface plasmon resonance spectroscopy binding analysis of sdAb to FGFR1, 2 and 3, Figure S6: SdAb-coupled liposomes on Rh4-FR4ko cells, Figure S7: Cytotoxicity of FGFR4 CAR T cells towards RMS cells, Table S1: Affinity measurements of sdAb was performed via surface plasmon resonance spectroscopy, Table S2: Affinity determination of sdAb A8 and B5 to FGFR2.
