# Supplementary material for: Novel FGFR4-Targeting Single-Domain Antibodies for Multiple Targeted Therapies against Rhabdomyosarcoma
Source: Cancers (Basel). 2020 Nov 10;12(11):3313. doi: 10.3390/cancers12113313 (PMC7696840; doi:10.3390/cancers12113313)
Supplement: Supplementary file 1 [file cancers-12-03313-s001.pdf]

# Supplementary Materials: Novel FGFR4-Targeting Single Domain Antibodies for Multiple Targeted Therapies Against Rhabdomyosarcoma

**Nagjie Alijaj, Sandrine Moutel, Zelia L. Gouveia, Maxim Gray, Maurizio Roveri, Dzhangar Dzhumashev, Florian Weber, Gianmarco Meier, Paola Luciani, Jochen K. Rössler, Beat W. Schäfer, Franck Perez and Michele Bernasconi**

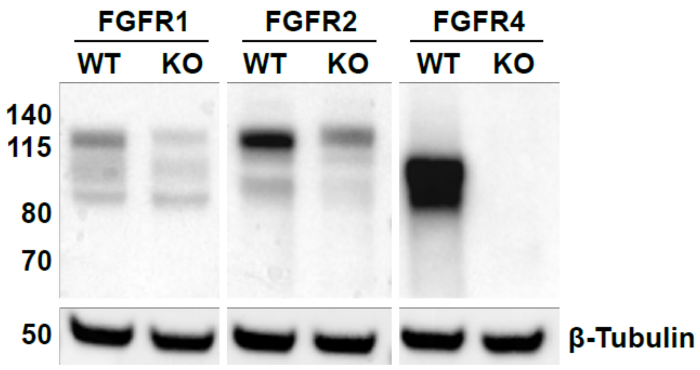


**Figure S1.** FGFR protein levels in Rh4 cells. Cells were transduced with a lentiviral vector containing Cas9 and sgRNA against FGFR4. Western blot analysis of Rh4-FR4wt (WT) and Rh4-FR4ko clone 8 (KO) were performed with antibodies against FGFR1, 2 and 4.

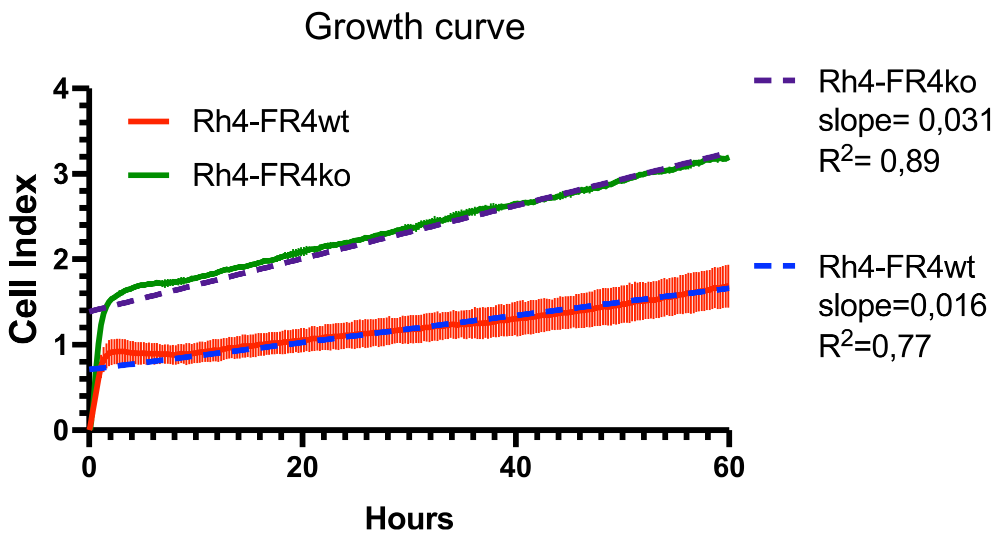


**Figure S2.** Real-time cell analysis of Rh4-FR4wt and Rh4-FR4ko cells growth. Rh4-FR4wt and Rh4-FR4ko cells were plated at 10,000 cell per well and let grow for 60 h. In this condition, the Rh4-FR4wt cells grown approximately twice shower than the KO cells, as indicated by the slope. The slope of the curve was determined using GraphPad Prism.

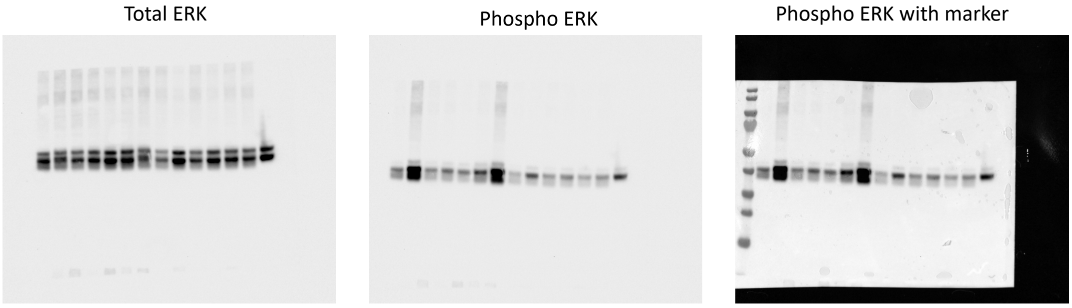


**Figure S3.** Original unmodified pictures of western blot shown in Figure 2C. Note that the first half of the samples (from the left) corresponds to Rh4-FR4wt and the other half to Rh4-FR4ko cells, which do not show any activation as expected.

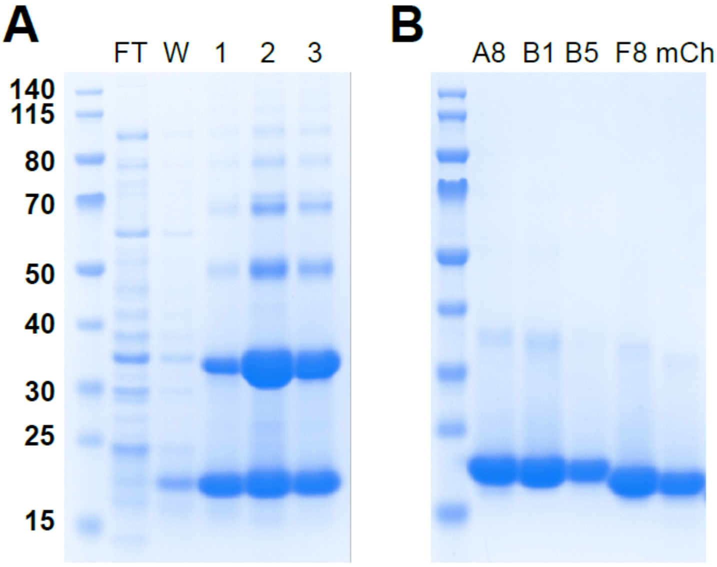


**Figure S4.** Purification of recombinantly expressed sdAb. (**A**) Flow-through and wash fraction of sdAb $Co^{2+}$ beads purification and three elution steps with 300 mM imidazole (elution 1–3). (**B**)**.** Size exclusion purified sdAb candidates and mCh negative control. Molecular weights are indicated on the left side in kDa.

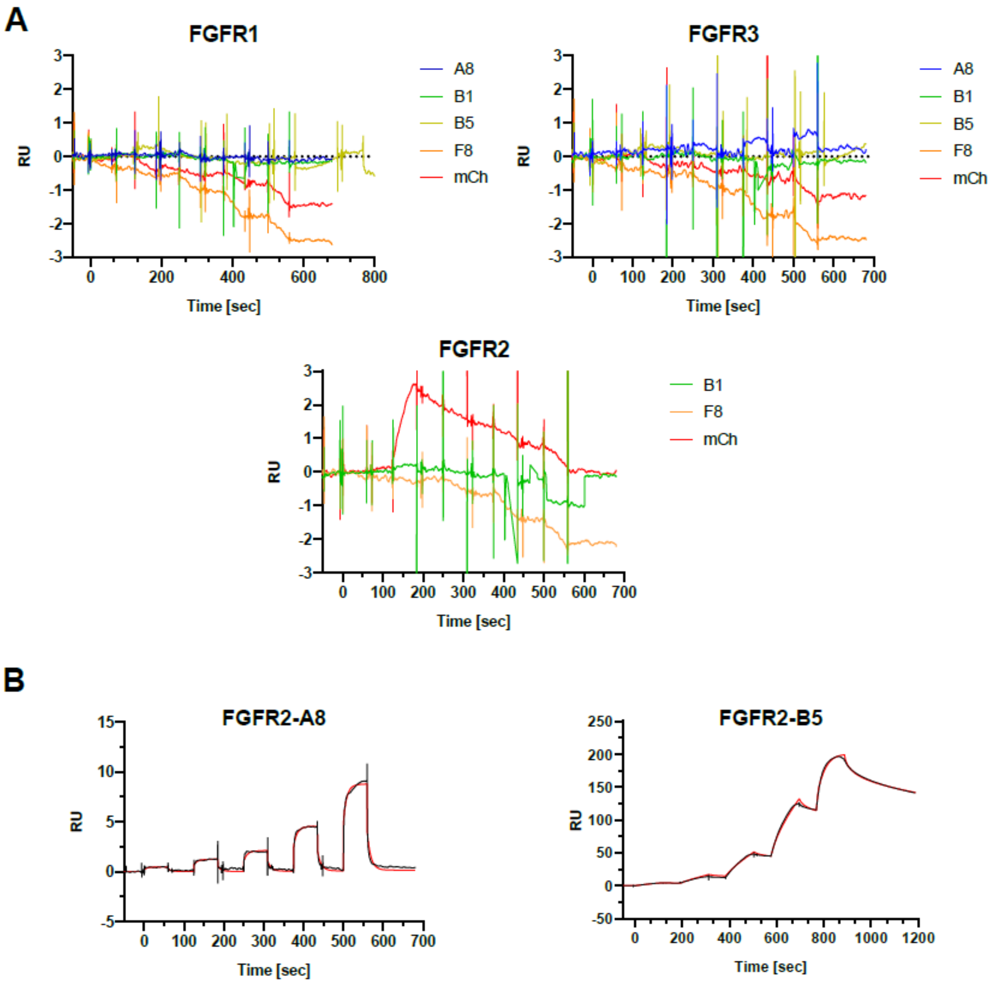


**Figure S5.** Surface plasmon resonance spectroscopy binding analysis of sdAb to FGFR1, 2 and 3. Single cycle kinetics measurements were performed with identical concentrations of sdAb used for affinity determination to FGFR4 by immobilizing recombinant FGFR1, 2 and 3 on a dextran based sensor chip. The analytes A8, B1, B5, F8 and mCh were injected in 5 different concentrations for the measurement of $k_{on}$ and $k_{off}$ rates. (**A**) Sensograms of non-binding sdAb to FGFR1, 2 and 3. (**B**). Candidates A8 and B5 bind to FGFR2. The black curves represent the measured data and red curves show the fit analysis (heterogeneous ligand model) performed with the BIAevaluation software.

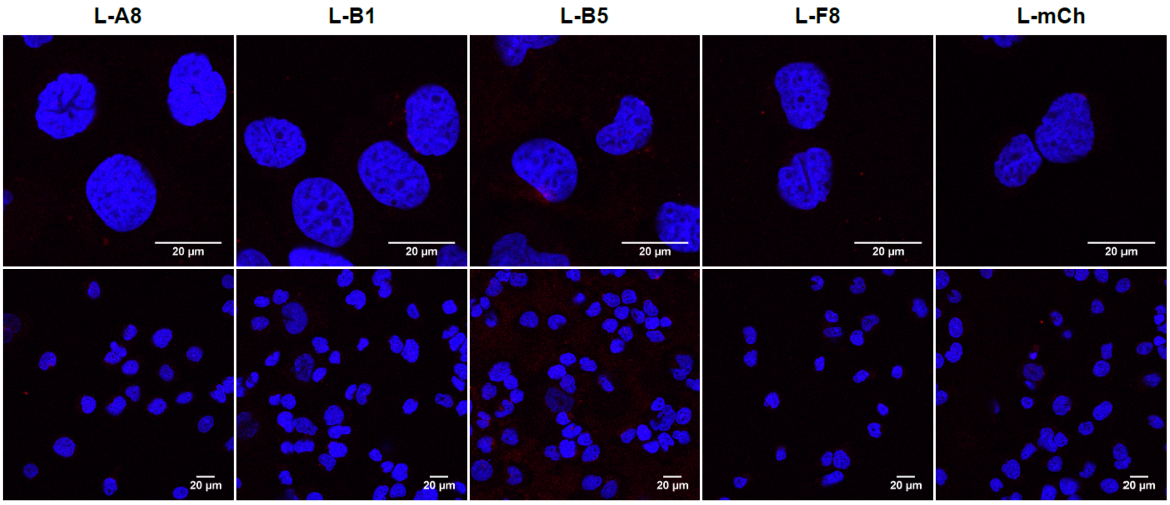


**Figure S6.** SdAb-coupled liposomes on Rh4-FR4ko cells. The cells were incubated for 2 h at 37 °C and 5% $CO_2$ with FGFR4-targeting and mCh control liposomes. The total lipid concentration was 3 mM. Cells were washed, fixed and mounted with DAPI containing medium.

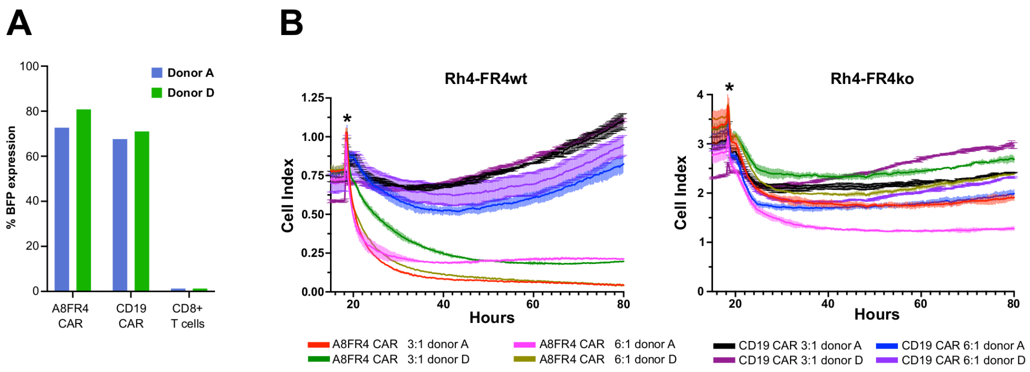


**Figure S7.** Cytotoxicity of FGFR4 CAR T cells towards RMS cells. (**A**) CD8+ T cell transduction efficiencies of donor A and D were determined by flow cytometry analysis of BFP signal. (**B**) Real-time cell death analysis of Rh4 cells co-cultured with effector T cells from donor A and D using xCELLigence RTCA DP. FGFR4 CAR T cells showed higher killing activities at the indicated E:T cell ratios in Rh4-FR4wt compared to non-specific CD19-CAR T cells. In Rh4-FR4ko cells no or reduced cytotoxicity was observed. The asterisks indicate the time of addition of the effector T cells.

**Table S1. Affinity measurements of sdAb performed via surface plasmon resonance spectroscopy.**

| SdAb | Asso (s) | Disso (s) | Final diss (s) | $C_1$ (M)* | $C_2$ (M) | $C_3$ (M) | $C_4$ (M) | $C_5$ (M) |
|---|---|---|---|---|---|---|---|---|
| **A8** | 120 | 70 | 500 | $2.80 \times 10^{-8}$ | $5.60 \times 10^{-8}$ | $1.13 \times 10^{-7}$ | $2.25 \times 10^{-7}$ | $4.50 \times 10^{-7}$ |
| **B1** | 240 | 70 | 650 | $6.25 \times 10^{-9}$ | $1.25 \times 10^{-8}$ | $2.50 \times 10^{-8}$ | $5.00 \times 10^{-8}$ | $1.00 \times 10^{-7}$ |
| **B5** | 180 | 70 | 300 | $6.30 \times 10^{-10}$ | $1.90 \times 10^{-9}$ | $5.60 \times 10^{-9}$ | $1.68 \times 10^{-8}$ | $5.00 \times 10^{-8}$ |
| **F8** | 60 | 60 | 120 | $3.40 \times 10^{-8}$ | $6.75 \times 10^{-8}$ | $1.35 \times 10^{-7}$ | $2.70 \times 10^{-7}$ | $5.40 \times 10^{-7}$ |
| **mCh** | 60 | 60 | 120 | $3.40 \times 10^{-8}$ | $6.75 \times 10^{-8}$ | $1.35 \times 10^{-7}$ | $2.70 \times 10^{-7}$ | $5.40 \times 10^{-7}$ |

*The analytes were injected in five different concentrations ($C_1$-$C_5$) for the indicated association time followed by a short dissociation phase. A final dissociation step was performed after the last and highest concentration injection.

**Table S2. Affinity determination of sdAb A8 and B5 to FGFR2.**

| SdAb | $k_{on}1$ (1/M*s) | $k_{off}1$ (1/s) | $K_D1$ (M) | $k_{on}2$ (1/M*s) | $k_{off}2$ (1/s) | $K_D2$ (M) | $R_{max}1$ (RU) | $R_{max}2$ (RU) |
|---|---|---|---|---|---|---|---|---|
| **A8** | $5.75 \times 10^{4}$ | $1.10 \times 10^{-1}$ | $1.91 \times 10^{-6}$ | $7.06 \times 10^{2}$ | $3.25 \times 10^{-5}$ | $4.60 \times 10^{-8}$ | 36.3 | 8.1 |
| **B5** | $7.88 \times 10^{4}$ | $5.94 \times 10^{-4}$ | $7.53 \times 10^{-10}$ | $1.04 \times 10^{10}$ | $5.74 \times 10^{1}$ | $5.54 \times 10^{-9}$ | 159.8 | 47.8 |

Surface plasmon resonance spectroscopy measurements and heterogeneous ligand binding fit revealed association- and dissociation constants (kon and koff) of the analytes for the immobilized recombinant protein. Binding affinities are expressed by the dissociation equilibrium constants KD (koff/kon) with respective maximal binding signals Rmax.

© 2020 by the authors. Licensee MDPI, Basel, Switzerland. This article is an open access article distributed under the terms and conditions of the Creative Commons Attribution (CC BY) license (http://creativecommons.org/licenses/by/4.0/).
